# The Psychological Impact of Vitiligo in Saudi Arabia

**DOI:** 10.7759/cureus.43767

**Published:** 2023-08-19

**Authors:** Mohammed Alkhowailed, Hatim M Alotaibi, Mohammed A Alshwieer, Alwaleed K Alazmi, Nawaf M Alotaibi, Abdulaziz F Alotaibi

**Affiliations:** 1 Dermatology, Qassim University, Buraydah, SAU; 2 General Practice, Ad-Dawadmi General Hospital, Ad-Dawadmi, SAU; 3 Medicine, College of Medicine, King Saud University Medical City, Riyadh, SAU; 4 Medicine, College of Medicine, Shaqra University, Ad-Dawadmi, SAU

**Keywords:** kingdom of saudi arabia (ksa), vitiligo, phq-9, dlqi, heath related quality of life

## Abstract

Background

Vitiligo, the most common pigment disorder, impacts 0.5-2% of the global population, often causing psychological distress due to appearance changes and potential discrimination. Existing data on depressive symptoms and quality of life (QoL) effects in Saudi Arabian vitiligo patients are limited and inconsistent. Hence, this multi-center investigation was conducted in Saudi Arabia to determine the prevalence of depressive symptoms and quality of life (QoL) impairment in patients with vitiligo and to identify factors linked to increased psychological distress in this population.

Methods

We conducted a multi-center cross-sectional study in Saudi Arabia, employing two validated Arabic questionnaires, the Dermatology Life Quality Index (DLQI) and the Patient Health Questionnaire-9 items (PHQ-9), along with demographic information. Appropriate statistical analyses were performed.

Results

In total, 204 patients completed the survey. The median DLQI was 4 (range: 25), while the median PHQ-9 was 5 (range: 27). Factors associated with worse QoL included an early age of onset (under 18 years) and a disease duration exceeding five years. Conversely, only disease duration correlated with worse PHQ-9 scores. Vitiligo lesions on the lower extremities, feet, and genitalia were linked to poorer DLQI scores, while only genitalia were associated with worse PHQ-9 scores. We recommend further social awareness campaigns emphasizing the role of supportive families to improve the well-being of vitiligo patients.

## Introduction

Vitiligo is a multifactorial pigmentary disorder in which melanocytes are progressively destroyed [1]. It is the most common depigmentary disorder, with a worldwide prevalence ranging from 0.5 to 2% [1,2].

Vitiligo has a significant psychological impact on patients due to appearance disfigurement. Patients with vitiligo often experience discrimination and are inclined to have low self-confidence, quality of life (QoL) impairment, and mental issues such as depression [3-5].

Skin diseases in Saudi Arabia showed significant differences in QoL scores, likely due to cultural differences in how people in Saudi Arabia experience skin diseases and view disabilities caused by them [6]. Patients with vitiligo in Middle Eastern countries are prone to experiencing more psychological stress compared to those in European countries. This discrepancy may be attributed to differences in skin type, the extent of public awareness, and social stigma [7].

A few articles have studied the psychological impact of vitiligo on patients in Saudi Arabia. The prevalence of depressive symptoms among these patients varies widely, ranging from 14% to 54.5% [8,9]. Moreover, the quality-of-life impairment in patients with vitiligo in Saudi Arabia was measured using the Dermatology Life Quality Index (DLQI) tool, and the studies revealed a wide variation in the level of impairment [10-12].

Previous literature on depressive symptoms and QoL impairment in patients with vitiligo in Saudi Arabia is scarce and highly varied. Therefore, we conducted this multi-centric study in central Saudi Arabia to measure the prevalence of depressive symptoms and QoL impairment in patients with vitiligo and to assess the factors associated with the worsening of psychological impairment in these patients.

## Materials and methods

Study design and settings

This cross-sectional study was conducted on 204 patients with vitiligo at the National Center of Vitiligo in Riyadh and Qassim University Medical City in the Qassim region of Saudi Arabia. The data were collected from February 2023 to April 2023. All the patients with vitiligo above 18 years visiting dermatology clinics of the aforementioned hospitals were included and asked to complete a survey using an Arabic version of the DLQI and Patient Health Questionnaire (PHQ-9). Patients were included if they were older than 18 years old and had a confirmed diagnosis of vitiligo by a dermatologist. Exclusion criteria included illiteracy, communication difficulties, or refusal to participate.

"All the patients with vitiligo above 18 years visiting dermatology clinics of the aforementioned hospitals were included."

Based on previous literature, we used 14% as the expected prevalence of depressive symptoms using the Kish 1956 Formula [8,13]. With 5% precision and a 95% confidence level, the estimated minimum required sample size was 185. The study was conducted after we gained ethical approval from the Institutional Review Board (IRB) of the College of Medicine, Qassim University, Qassim, Saudi Arabia.

Questionnaire content

The questionnaire consists of five sections: A) informed consent; B) socio-demographic characteristics, which include age, gender, marital status, and educational level; C) disease information, which includes skin variants, disease duration, site of affection, the onset of the disease, and family history; D) Arabic version of the DLQI; E) Arabic version of the PHQ-9

Quality of life measurement tool

We used a validated Arabic DLQI questionnaire to assess the patient's perception of the impact of skin diseases on various aspects of their health-related quality of life over the last week. The questionnaire consists of 10 questions, with each question scoring from zero to three. The total DLQI score is classified into five levels: 0-1= no effect, 2-5= small effect, 6-10= moderate effect, 11-20= very large effect, and 21-30= extremely large effect [14,15]. We obtained permission to use the Arabic version for the purpose of this study.

Depression measurement tool

We used a validated Arabic PHQ-9 questionnaire to assess the degree of depression present in an individual. The questionnaire consists of nine questions, with each question scoring from zero to three. The total PHQ-9 score is classified into four levels: 0-4= minimal depression, 5-9= mild depression, 10-14= moderate depression, 15-20= moderately severe depression, and 21-27= severe depression [16,17]. A PHQ-9 score ≥9 is considered the optimal cut-off score for depressive symptoms [16]. PHQ-9 is available and free for non-commercial users.

Statistical analyses

We used IBM SPSS Statistics for Windows, Version 26.0 (released 2019; IBM Corp., Armonk, New York, United States) to analyze the data in this study. The categorical variables are presented as frequencies and percentages. Data normality testing was performed using the Shapiro-Wilk test. The continuous variables with an abnormal distribution are presented with medians and ranges. The comparison between variables’ medians was performed using the Student’s t-test. Statistical significance is determined with a P-value < 0.05 and 95% confidence intervals.

## Results

**Figure 1:**
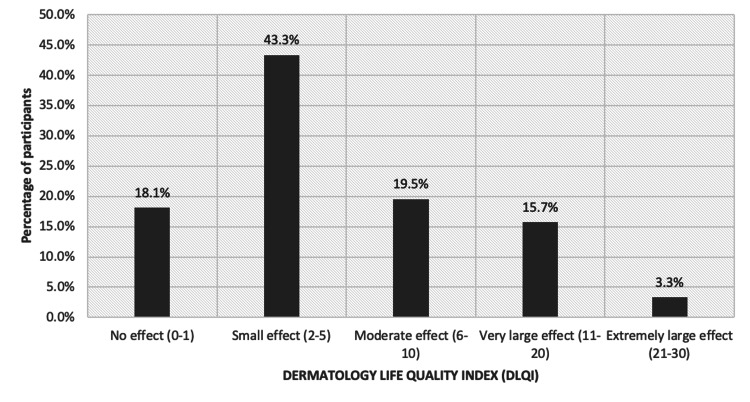
The percentage of the patients according to the DLQI Scores

**Figure 2:**
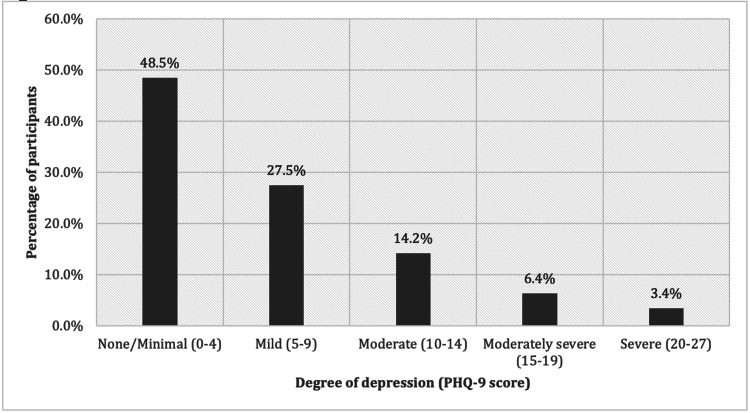
The percentage of the patients according to the PHQ-9 scores

A total of 204 vitiligo patients completed the survey. Almost half of the patients were younger than 30 years old (52.9%), female (55.9%), and single (52.0%). The majority of the patients were highly educated (91.7%). Approximately two-thirds of the patients had dark skin (67.6%). Other characteristics are shown in Table 1. The percentage of patients according to the DLQI classification is shown in Figure 1. The percentage of patients according to the PHQ-9 classification is shown in Figure 2.

**Table 1:** Demographic data and disease characteristics by median DLQI and PHQ-9 scores (N = 204) The DLQI score median is 4, the range 25, minimum 0, maximum 25, skewness 1.373, and kurtosis 1.572. The PHQ-9 score median is 5, the range 27, minimum 0, maximum 27, skewness 1.044, and kurtosis 0.536. -ve= negative, +ve= positive *Statistically significant P < 0.05

		DLQI		PHQ-9	
Variables	n (%)	MEDIAN (range)	P-value	MEDIAN (range)	P-value
Age group					
Less than 30 years	108 (52.9)	4 (25)	0.386	5 (27)	0.554
30 years and older	96 (47.1)	4 (20)		4 (20)	
Gender					
Male	90 (44.1)	4 (20)	0.749	4 (21)	0.409
Female	114 (55.9)	4 (25)		5 (27)	
Marital status					
Married	98 (48.0)	5 (20)	0.889	5 (20)	0.225
Single	106 (52.0)	4 (25)		5 (27)	
Education level					
High school or lesser	17 (8.3)	3 (20)	0.325	3 (17)	0.176
University or higher	187 (91.7)	4 (25)		5 (27)	
Skin type					
Fair skin	66 (32.4)	4 (25)	0.113	5 (20)	0.668
Dark skin	138 (67.6)	4 (20)		5 (27)	
Disease duration					
Five years and less	53 (26.0)	3 (19)	0.020*	4 (16)	0.200
More than five years	151 (74.0)	5 (25)		5 (27)	
Age of onset					
< 18 years old	115 (56.4)	5 (25)	0.028*	5 (27)	0.024*
≥18 years old	89 (43.6)	3 (20)		4 (17)	
Family history of vitiligo					
-ve Family history	128 (62.7)	4 (25)	0.301	5 (21)	0.811
+ve Family history	76 (37.3)	4 (17)		4 (27)	

The Shapiro-Wilk test was used to test the normality of the distribution of the DLQI and the PHQ-9 scores. The data for both scores did not meet the normality assumption; therefore, the median was used for further analysis. Table 1 shows the results: the median DLQI score was 4, the range was 25, minimum 0, maximum 25, skewness was 1.373, and kurtosis was 1.572. The median PHQ-9 score was 5, the range was 27, minimum 0, maximum 27, skewness 1.044, and kurtosis 0.536.

The association between the DLQI and the PHQ-9 scores with demographic and disease characteristics is presented in Table 1. The median DLQI score was statistically significantly higher in patients with a disease duration of more than five years and age of onset less than 18 years (P < 0.05). The median PHQ-9 was statistically significantly higher in patients with an age of onset of less than 18 years (P < 0.05). Disease duration was not statistically significantly associated with a higher median PHQ-9 (P > 0.05). Other variables in the model (age, gender, marital status, education level, skin type, and family history) did not show a significant difference in the median DLQI and PHQ-9 scores (P > 0.05).

**Figure 3:**
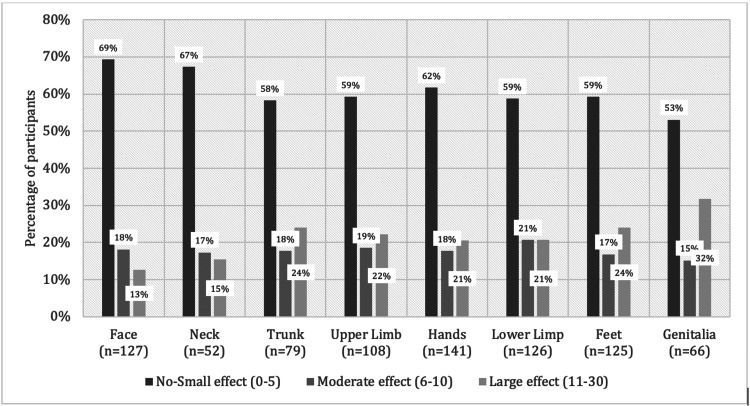
Correlation between skin involvement and Dermatology Quality Life Index (DLQI) among vitiligo patients

**Figure 4:**
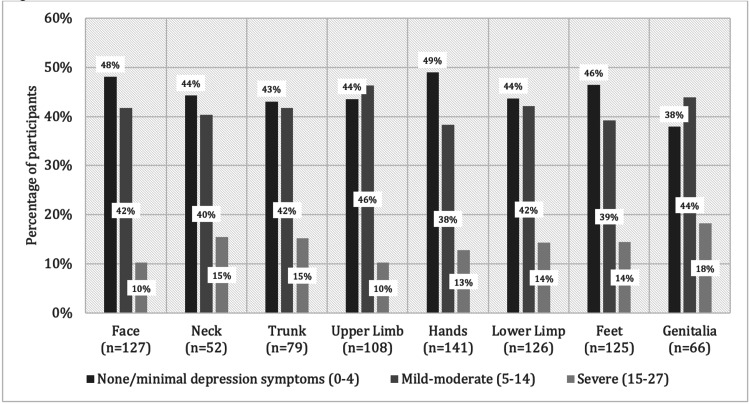
Correlation between skin involvement and severity of depression according to Patient Health Questionnaire-9 (PHQ-9) among vitiligo patients

Table 2 describes the association of DLQI and PHQ-9 scores with vitiligo-involved areas, namely the face, neck, upper extremities, trunk, lower extremities, hands, feet, and genitalia. The median DLQI score was statistically significantly higher in patients with involvement of the hands, lower extremities, feet, and genitalia than in patients without face involvement (P < 0.05). The median PHQ-9 score was statistically significantly higher only in patients with genitalia involvement (P < 0.05). Figure 3 demonstrates the percentage of patients according to areas involved with vitiligo and the DLQI classification. Figure 4 demonstrates the percentage of patients according to areas involved with vitiligo and the PHQ-9 classification.

**Table 2:** Vitiligo affected areas by median DLQI and PHQ-9 scores (N = 204) *Statistically significant P < 0.05

		DLQI		PHQ-9	
Variables	n (%)	MEDIAN (range)	P-value	MEDIAN (range)	P-value
Face					
No	77 (37.7)	5 (20)	0.048*	5 (21)	0.507
Yes	127 (62.3)	4 (25)		5 (27)	
Neck					
No	152 (74.5)	4 (20)	0.726	4.5 (21)	0.947
Yes	52 (25.5)	4 (25)		5.5 (27)	
Trunk					
No	125 (61.3)	4 (19)	0.080	4 (21)	0.554
Yes	79 (38.7)	5 (25)		5 (27)	
Upper extremities					
No	96 (47.1)	4 (20)	0.108	4 (27)	0.288
Yes	108 (52.9)	5 (25)		5 (21)	
Lower extremities					
No	78 (38.3)	3 (21)	0.003*	4 (16)	0.071
Yes	126 (61.8)	5 (27)		5 (25)	
Hands					
No	63 (30.9)	4 (15)	0.499	5 (27)	0.983
Yes	141 (69.1)	4 (25)		5 (21)	
Feet					
No	79 (38.7)	4 (15)	0.042*	4 (21)	0.240
Yes	125 (61.3)	5 (25)		5 (27)	
Genitalia					
No	138 (67.6)	4 (20)	0.020*	4 (21)	0.001*
Yes	66 (32.4)	5 (25)		7.5 (27)	

## Discussion

The results of our study help to clarify the psychological impact of vitiligo on patients in Saudi Arabia. The prevalence of depressive symptoms based on the PHQ-9 ≥ 9 cut-off among our patients was 30.9%. The impact of vitiligo on patients’ QoL was small to moderate based on the mean DLQI score (5.58 ± 4.9 SD), which we used for the sake of comparison to the other studies.

Globally, the prevalence of depressive symptoms among vitiligo patients ranges from 0.1% to 62.3% with a pooled prevalence of 33% which is close to our result [18,19]. Regarding QoL, recent systematic literature reviews showed a global DLQI range of 1.82 to 15.0. QoL is least impaired in European and North American countries and most impaired in Middle Eastern and Asian countries [19,20].

In Saudi Arabia, the prevalence of depressive symptoms among vitiligo patients has experienced a series of ups and downs over the years. Prevalence rose from 14% in 2018 to 54.5% in 2020 before dropping again to 30.9% in our study [8,9]. Interestingly, these fluctuations occurred despite using different scales for measurement.

Our findings are consistent with previous studies that assessed the prevalence of depression in the Saudi population. Specifically, the prevalence rate of 18.14% obtained in this study using a PHQ-9 cut-off score of ≥12 is comparable to the 14% reported in Alshahwan's study that employed a Hospital Anxiety and Depression Scale (HADS-D) cut-off of ≥10 [8]. This HADS-D cut-off tends to under-include compared to the PHQ-9 score ≥9 [21]. Hansson et al. suggested using HADS ≥8 and PHQ-9 ≥12 cut-offs to achieve comparable prevalence rates with different scales [22]. In contrast, the 54.5% prevalence reported in Alharbi's study used an unusually low Beck Depression Inventory (BDI) cut-off score of ≥5. This cut-off is substantially lower than the suggested comparable cut-off of ≥14 in the BDI to the ≥10 in the PHQ-9. Therefore, the high prevalence rate found in Alharbi's study should be interpreted with caution due to the low cut-off used, which likely led to an overestimation of depression prevalence. The choice of cut-off score has a significant impact on prevalence estimates [9,23]. Furthermore, the PHQ-9 score is more directed toward the Diagnostic and Statistical Manual of Mental Disorders (DSM) Criteria for Major Depressive Episodes compared to the BDI score and thus is more reliable for depression scaling [24].

In Saudi Arabia, vitiligo seems to have a deleterious impact on QoL as well. The first study to utilize the DLQI in Saudi Arabia was in 2007. This study showed that the QoL impairment score was (14.72 ± 5.173 SD) [12]. Granted, in 2013 and 2015, the score decreased to (9 ± 6.5 SD) and (10.6 ± 4.3) respectively. In 2021, the score was 5.64 ± 5.2 SD, which is similar to our results [10,11,25]. This improvement in QoL is likely due to the intensive social education and public awareness campaigns, which have expanded education levels among patients and in society as a whole and alleviated the burden on vitiligo patients.

While the overall QoL of vitiligo patients in Saudi Arabia has improved in recent years, significant disparities between some demographic groups persist. Our study found that age of onset had a significant impact on both QoL and psychological health, with those who developed vitiligo at a younger age experiencing more severe effects on their QoL and more depressive symptoms. This finding is consistent with a systematic review showing that earlier disease onset was associated with greater QoL impairment [3]. This may be because adolescence is a critical period for the development of self-esteem and identity, and younger patients may be more sensitive to body image and peer pressure [26,27].

Disease duration (>five years) was associated with significantly higher QoL impairment but no difference in depressive symptoms. This is consistent with a recent systematic review result [19]. Longer disease duration is traced to longer periods of embarrassment, social isolation, and lower hope of getting off the disease [27].

A recent systematic review identified factors that contribute to lower QoL among vitiligo patients. Being female and younger than 30 years old was among the most frequent factors in these studies [20]. Nevertheless, our results didn’t reveal any association between these factors and our outcomes. In fact, the insignificance of these specific factors within our community highlights the crucial role played by the family unit in Saudi society. An emotionally and psychologically supportive family is especially beneficial for vulnerable individuals, such as women and adolescents. This discovery reinforces the importance of families and the broader community in helping individuals with vitiligo adapt to their condition [28].

In our study, patients without face involvement had significantly lower QoL than patients with face involvement. This interesting finding contradicts the majority of previous studies, which suggest that exposed area involvement, especially the face, predicts higher QoL impairment [10,19,20,29]. This finding could be attributed to the fear of developing facial involvement in patients who don't yet have it, while patients with facial involvement have already adapted to the "worst" of vitiligo.

We found that patients with genital involvement had a significant association with poorer QoL and more depressive symptoms. This may be attributed to the patient's shame and fear of being rejected by their partner. Furthermore, it has been reported that genital lesions play a major role in developing sexual dysfunction in vitiligo patients [3,19,30].

Limitations

Since this is a cross-sectional study, the results only show a correlation between variables, not causation. Also, this study relied on the DLQI, which can help estimate the overall QoL in patients with dermatological diseases but can’t show the burden of vitiligo in detail.

## Conclusions

We conclude in this study that vitiligo has a substantial negative psychological impact on patients with vitiligo in Saudi Arabia, with an alarming prevalence of depressive symptoms of 30.9% as per the PHQ-9 questionnaire. Particularly noteworthy was the correlation between genital involvement and a younger age of onset with a heightened severity of depressive symptoms and QoL impairment. Intriguingly, the study also underscored that patients with longer disease duration and without facial involvement exhibited worse QoL.

Although having a negative impact, both the quality of life and depressive symptoms scores have improved over the years in Saudi Arabia, likely due to increased awareness and public education. We recommend further social awareness campaigns emphasizing the role of supportive families to improve the well-being of vitiligo patients.
